# Efficacy of Vitamin E Administration on Serum Lipid Profile in Adult Patients With Dyslipidemia: A GRADE‐Assessed Systematic Review and Dose–Response Meta‐Analysis of RCTs

**DOI:** 10.1002/fsn3.72428

**Published:** 2026-10-07

**Authors:** Mehdi Karimi, Kimia Kazemi, Fatemeh Ziyafati, Fereshteh Valizadeh, Niyousha Shirsalimi, Sepehr Ramezanipour, Ali Estifa, Omid Asbaghi

**Affiliations:** ^1^ Faculty of Medicine Bogomolets National Medical University (NMU) Kyiv Ukraine; ^2^ Department of Food Science and Technology Islamic Azad University—Ayatollah Amoli Branch Amol Iran; ^3^ Department of Genetics, Reproductive Biomedicine Research Center Royan Institute for Reproductive Biomedicine, ACECR Tehran Iran; ^4^ Faculty of Medicine Shahid Beheshti University of Medical Sciences (SBUMS) Tehran Iran; ^5^ Faculty of Medicine Hamadan University of Medical Sciences (UMSHA) Hamadan Iran; ^6^ Student Research Committee Fasa University of Medical Sciences Fasa Iran; ^7^ Department of Sport Sciences Imam Khomeini International University Qazvin Iran; ^8^ Cancer Research Center Shahid Beheshti University of Medical Sciences (SBUMS) Tehran Iran

**Keywords:** blood lipids, cardiovascular disease, dyslipidemia, hyperlipidemia, nutritional supplementation, tocopherol, tocotrienol, vitamin E

## Abstract

Dyslipidemia is a major risk factor for cardiovascular disease. Studies have shown that vitamin E has therapeutic effects on lipid metabolism; however, findings remain inconsistent. This meta‐analysis aims to assess the effectiveness of vitamin E administration on the serum lipid panel in patients with dyslipidemia. A database search through January 2026 was conducted to identify relevant randomized controlled trials (RCTs). Relevant data, including triglycerides (TG), total cholesterol (TC), and low‐ and high‐density lipoprotein cholesterol (LDL‐C and HDL‐C), were extracted. The overall effect size was estimated using weighted mean differences (WMDs) with 95% confidence intervals (CIs) via a random‐effects model. The pooled analysis of 22 RCTs (33 intervention arms) demonstrated that vitamin E significantly lowered TG (WMD: −8.14 mg/dL, *p* = 0.021) and raised HDL‐C (WMD: 1.08 mg/dL, *p* = 0.037) in patients with dyslipidemia, whereas reductions in TC (WMD: −6.48 mg/dL, *p* = 0.097) and LDL‐C (WMD: −9.06 mg/dL, *p* = 0.063) were not significant overall. Exploratory subgroup analyses suggested that tocotrienols may exert greater lipid‐modifying effects than tocopherols, with potentially larger benefits observed at lower doses, among individuals with more pronounced baseline lipid abnormalities, and during shorter intervention periods. In conclusion, vitamin E supplementation in patients with dyslipidemia shows modest improvements in lipid profiles, mainly lowering TG and increasing HDL‐C, with less consistent effects on TC and LDL‐C. Tocotrienols may be more effective than tocopherols, suggesting possible isoform‐specific effects, but overall clinical impact appears limited and requires further confirmation.

## Graphical abstract

The graphical abstract summarizes a GRADE‐assessed systematic review and dose–response meta‐analysis of 22 randomized controlled trials evaluating oral vitamin E supplementation in adults with dyslipidemia. It presents the effects on serum lipid parameters, including triglycerides (TG), total cholesterol (TC), LDL‐C, and HDL‐C. Vitamin E significantly reduced TG by 8.14 mg/dL and increased HDL‐C by 1.08 mg/dL, while reductions in TC and LDL‐C were not statistically significant.

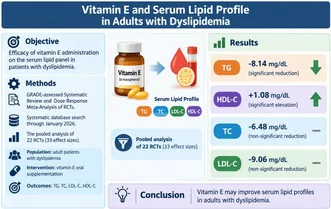

**Abbreviations**
HDL‐C: high‐density lipoprotein cholesterol
LDL‐C: low‐density lipoprotein cholesterol
PCOS: polycystic ovary syndrome
RCT: randomized controlled trial
TC: total cholesterol
TG: triglycerides

## 1 Introduction

Dyslipidemia is a metabolic problem characterized by abnormal and imbalanced levels of serum lipids and is a major risk factor for atherosclerotic cardiovascular disease mortality worldwide (Sandesara et al. 2019). Genetic and environmental factors influence the epidemiology of dyslipidemia and vary by region, age, sex, and ethnicity (Pirillo et al. 2021). According to the latest research, the global prevalence of dyslipidemia in adults is estimated to be between 20% and 80%, depending on the definition and criteria used (Mohamed‐Yassin et al. 2021). Management of dyslipidemia generally entails a combination of lifestyle changes, such as dietary adjustments, regular physical activity, weight control, and smoking cessation, along with pharmacological treatments, including statins and fibrates (Pappan et al. 2024). In recent years, there has been increasing interest in complementary and alternative approaches to dyslipidemia management, particularly the use of dietary supplements that may favorably influence lipid metabolism and promote cardiovascular health (Karimi et al. 2024; Hamednia et al. 2025; Karimi, Javadi, et al. 2025; Karimi, Asbaghi, et al. 2026). Among these supplements, vitamin E has attracted considerable attention due to its potential lipid‐modifying properties and its supportive role in cardiovascular function (Karimi, Karimi, et al. 2025; Karimi, Abdi, et al. 2026; Karimi, Alipour, et al. 2025).

Vitamin E refers to a family of fat‐soluble compounds, primarily tocopherols and tocotrienols, that exhibit potent antioxidant activity, each with distinct biological functions (Jiang 2022). Tocotrienols differ structurally from tocopherols in that they possess an unsaturated isoprenoid side chain, which enhances their mobility and incorporation into lipid membranes. This structural characteristic may confer superior antioxidant capacity and distinct metabolic effects compared with tocopherols. In addition to their antioxidant properties, tocotrienols have been shown to exert cholesterol‐lowering effects through posttranscriptional suppression of HMG‐CoA reductase, the rate‐limiting enzyme in cholesterol biosynthesis, providing a mechanistic basis for lipid modulation beyond free radical scavenging (Colombo 2010). Vitamin E plays a pivotal role in shielding lipoproteins from oxidative stress, a major factor in the onset of atherosclerosis and other cardiovascular disorders (Jiang 2022; Fatima et al. 2026). By embedding itself within the hydrophobic core of cell membranes, vitamin E safeguards membrane integrity through electron donation to lipid peroxidation intermediates, effectively neutralizing free radicals (Kilicarslan You et al. 2024). Evidence from both in vitro and in vivo studies indicates that vitamin E mitigates lipid peroxidation by disrupting the propagation phase of oxidation, thereby improving the oxidative stability of LDL‐C (Niki 2014). Collectively, these antioxidant and lipid‐regulatory mechanisms underscore the potential cardioprotective role of vitamin E, particularly in individuals with dyslipidemia.

Multiple studies have examined the potential association between vitamin E supplementation and blood lipid levels across populations (Zuo et al. 2020; Krasinski et al. 1989). Despite this, the relationship remains unclear, with findings showing considerable variability. For instance, a meta‐analysis of 15 trials involving 931 participants with diverse health conditions reported that intake of over 200 mg/day of vitamin E was associated with notable improvements in serum triglyceride (TG) and high‐density lipoprotein cholesterol (HDL‐C) levels (Zuo et al. 2020). Conversely, another meta‐analysis of hemodialysis patients found no significant effect of vitamin E on lipid profiles (Huang et al. 2015). Furthermore, research investigating the influence of dosage and duration suggests that combining 250 mg/day and 400 IU/day of vitamin E for at least 1.5 months can significantly enhance serum TG, total cholesterol (TC), and low‐density lipoprotein cholesterol (LDL‐C) levels in individuals with polycystic ovary syndrome (PCOS) or gestational diabetes (Dehbalaei et al. 2021).

Despite several previous meta‐analyses evaluating vitamin E supplementation, important evidence gaps remain. Existing reviews have primarily included heterogeneous populations with diverse metabolic disorders or focused on specific diseases such as diabetes mellitus, limiting their applicability to patients with established dyslipidemia. Furthermore, previous studies generally did not comprehensively evaluate vitamin E isoforms, dose–response relationships, or the certainty of evidence using the GRADE framework. Therefore, this study was designed as the first GRADE‐assessed dose–response meta‐analysis conducted exclusively in adults with dyslipidemia, incorporating recently published RCTs and separately evaluating tocopherol and tocotrienol isoforms to provide more clinically relevant evidence for this patient population.

## 2 Method

### 2.1 Study Design and Protocol

This study was conducted in accordance with the PRISMA statements (Preferred Reporting Items for Systematic Reviews and Meta‐Analyses) (Page et al. 2021). The study was framed based on the PICOS (Population, Intervention, Comparison, Outcomes, and Study) framework (Amir‐Behghadami and Janati 2020), including:

– P (Population): Adult patients with dyslipidemia.
– I (Intervention): Oral supplementation of vitamin E, or its isoforms, including tocopherols and tocotrienols (α, β, γ, δ forms).
– C (Comparison): Placebo, no intervention, standard care, or active comparator(s), provided that any co‐interventions (e.g., statins or gemfibrozil) were administered equally in both study arms, with vitamin E supplementation representing the only difference between the intervention and control groups.
– O (Outcomes): Changes in serum lipid profile, including TG, TC, LDL‐C, and HDL‐C.
– S (Study Design): RCT.

### 2.2 Literature Search Strategy

A systematic search was conducted across major medical databases, including MEDLINE/PubMed, ISI Web of Science, and Scopus, to identify RCTs evaluating the effects of vitamin E on serum lipid profiles in patients with dyslipidemia. Studies published in English were considered, with no restriction on publication date, and the search encompassed all relevant literature available up to 01 January 2026. Both Medical Subject Headings (MeSH) and free‐text terms were employed to maximize search sensitivity, targeting titles and abstracts and utilizing Boolean operators (AND, OR) to refine results. the search keywords included (“Vitamin E” OR “Tocopherol” OR “tocotrienol” AND “Triglyceride” OR “Triacylglycerol” OR “cholesterol” OR “Lipoprotein” OR “low‐density lipoprotein” OR “LDL” OR “LDL‐C” OR “VLDL” OR “high‐density lipoprotein” OR “HDL” OR “HDL‐C” OR “Lipid profile” OR “lipid panel” AND “Dyslipidemi*” OR “hyperlipidemi*” OR “hypolipidemi*” OR “hypertriglyceridemi*” OR “hypotriglyceridemi*” OR “hypercholesterolemi*” OR “hypocholesterolemi*” OR “hypolipoproteinemi*” OR “hyperlipoproteinemi*”). To ensure comprehensiveness, reference lists of relevant studies were also manually screened.

### 2.3 Eligibility Criteria

Studies were eligible for inclusion if they met all of the following criteria: (1) employed a RCT design; (2) enrolled adult participants diagnosed with dyslipidemia; (3) investigated oral vitamin E supplementation (including tocopherol or tocotrienol isoforms) as the primary intervention; (4) included a placebo, no‐treatment, standard‐care, or active‐comparator control group, provided that any concomitant interventions (e.g., statins, fibrates, or other lipid‐lowering medications) were administered equally in both the intervention and control groups, such that vitamin E supplementation represented the only difference between study arms; (5) reported quantitative data on at least one lipid profile parameter, including TG, TC, LDL‐C, or HDL‐C; and (6) provided sufficient statistical information at baseline and post‐intervention, or change‐from‐baseline data, to enable effect size estimation.

Studies were excluded if they met any of the following criteria: (1) were non‐randomized studies, observational studies, case reports, case series, reviews, or conference abstracts without sufficient data; (2) evaluated Vitamin E as part of combination therapy in which the independent effect of vitamin E could not be isolated because concomitant interventions differed between study arms; (3) included pediatric populations or participants without dyslipidemia; (4) lacked an appropriate control group; (5) did not report relevant lipid profile outcomes (TG, TC, LDL‐C, or HDL‐C); or (6) provided insufficient quantitative data for extraction or statistical analysis.

### 2.4 Data Extraction

Data extraction was performed independently by two reviewers (O.A. and F.V.), with discrepancies resolved through discussion or consultation with a third reviewer (M.K.). The following information was extracted from each eligible study: study characteristics (first author, publication year, study design, sample size, age range, and gender distribution), intervention details (form, dosage, and route of vitamin E administration), and comparator (placebo or control group). Vitamin E isoforms were categorized according to the chemical form explicitly reported in each study. Trials administering α‐, β‐, γ‐, or δ‐tocopherol were classified as tocopherol studies, whereas those using tocotrienol‐rich fractions or isolated tocotrienol isomers were categorized as tocotrienol interventions. Subgroup analyses were performed accordingly. Outcomes, including baseline and posttreatment values for TG, TC, LDL‐C, and HDL‐C, were extracted, along with changes in lipid profiles and corresponding statistical measures (mean, standard deviation, *p* values, and confidence intervals). All extracted data were systematically entered into a standardized Excel sheet to ensure consistency.

### 2.5 Quality Assessment

The quality assessment of the RCTs was conducted independently by two reviewers (M.K. and F.V.) using the Cochrane Risk of Bias 2 (RoB‐2) tool. The assessment followed version 2 of the RoB‐2 tool to evaluate the quality of all eligible studies across five key domains: bias arising from the randomization process (D1), deviations from the intended intervention (D2), missing outcome data (D3), accuracy in measuring outcomes (D4), and selection of reported results (D5). Each domain was classified as “Low risk,” “High risk,” or “Some concerns” for risk of bias (Flemyng et al. 2023).

### 2.6 Certainty of Evidence

The certainty of the evidence was evaluated using the GRADE (Grading of Recommendations, Assessment, Development, and Evaluation) framework, which systematically assesses factors such as risk of bias, inconsistency, indirectness, imprecision, and potential publication bias. Each outcome was rated on a four‐tier scale of certainty: high, moderate, low, or very low (Hozo et al. 2005). This assessment involved a careful review of study design, methodological rigor, and the overall robustness of the evidence, ensuring a comprehensive and transparent appraisal of the findings (Flemyng et al. 2023). For outcomes informed by trials that contributed multiple intervention arms or by crossover designs, the risk‐of‐bias domain was evaluated with additional attention to the statistical independence of pooled comparisons and to the adequacy of the methods used to account for within‐person correlation, consistent with Cochrane Handbook guidance on GRADE assessment of complex evidence structures.

### 2.7 Statistical Analyses

Statistical analyses were performed using Stata version 11.1 (StataCorp, College Station, TX, USA), with a significance level set at *p* < 0.05 for all two‐tailed tests. A random‐effects model was used to estimate the pooled weighted mean difference (WMD), adjusting for heterogeneity (DerSimonian and Laird 1986). Mean differences (MD) in outcomes were calculated between baseline and post‐intervention values for both the intervention and control groups. The standard deviation (SD) of mean differences was computed using the formula: SD = √[(SD at baseline)^2^ + (SD at the end)^2^ − (2 × *r* × SD at baseline × SD at the end)], using an assumed within‐group correlation coefficient of *r* = 0.8, a value commonly adopted in meta‐analyses when individual participant data are unavailable (Setayesh et al. 2026; Papadopoulos 2025). For studies reporting standard errors (SEs) rather than SDs, the method of Hozo et al. (2005) was used to convert SEs, 95% confidence intervals (CIs), and interquartile ranges to SDs.

For crossover trials, paired within‐person data were analyzed using the same effect size framework as parallel‐group trials. The variance of paired comparisons was estimated using the standard deviation of the difference, assuming a within‐person correlation coefficient of *r* = 0.8, consistent with recommended approaches when individual participant data are unavailable (DerSimonian and Laird 1986; Setayesh et al. 2026). Participants were included in both intervention and control comparisons but counted only once in the overall sample size to avoid double‐counting. Adequate washout periods were considered when assessing potential carryover effects. The robustness of pooled estimates to the inclusion of crossover trials was evaluated using leave‐one‐out sensitivity analyses.

Subgroup analyses were performed using predefined cutoffs based on clinically recognized guideline thresholds and the distribution of the included studies. Baseline lipid categories were defined according to established diagnostic criteria for dyslipidemia, whereas BMI categories were defined according to the World Health Organization (WHO) classification. Intervention duration (< 12 vs. ≥ 12 weeks) reflected the most common treatment duration across the included RCTs, and the vitamin E dose threshold (≤ 400 vs. > 400 IU/day) was selected based on its frequent use in prior RCTs and meta‐analyses.

Heterogeneity was evaluated using the *I* ^2^ statistic. According to Cochrane recommendations, *I* ^2^ values of 0%–40% may not be important, 40%–60% may represent moderate heterogeneity, 60%–80% may represent substantial heterogeneity, and 80%–100% may represent considerable heterogeneity. Publication bias was evaluated through visual inspection of funnel plots and by conducting Egger's and Begg's tests, with sensitivity analyses performed using the leave‐one‐out method (Begg and Mazumdar 1994; Egger et al. 1997). Meta‐regression was used to examine the effects of intervention dose and duration on outcomes, and the trim‐and‐fill method was applied to address publication bias (Duval 2005).

## 3 Results

### 3.1 Study Selection Process

The study selection process commenced with the identification of 5155 records from major scientific databases: MEDLINE/PubMed (*n* = 709), ISI Web of Science (*n* = 1607), and Scopus (*n* = 2839). After removing duplicates, 3526 records remained and were initially screened based on titles and abstracts. Of these, 3486 were excluded because they were review articles, nonhuman studies (in vitro, in vivo, in situ, etc.), case reports, letters, opinions, or otherwise irrelevant. The full texts of 40 articles were then evaluated for eligibility; 18 were excluded for failing to meet inclusion criteria, such as lack of randomization, absence of a control group, or insufficient data. Ultimately, 22 RCTs (Leonhardt 1978; Qureshi et al. 1991, 1997, 2002; Wahlqvist et al. 1992; McDowell et al. 1994; Tomeo et al. 1995; Chowienczyk et al. 1998; Mensink et al. 1999; Kugiyama et al. 1999; Heitzer et al. 1999; O'Byrne et al. 2000; Kaikkonen et al. 2000; Borovničar et al. 2000; Mustad et al. 2002; Pereira et al. 2004; Baliarsingh et al. 2005; Sutken et al. 2006; Leonard et al. 2007; Zaiden et al. 2010; Tabaei et al. 2020; Namayandeh et al. 2007) were included in the systematic review and meta‐analysis (Figure 1).

**FIGURE 1:**
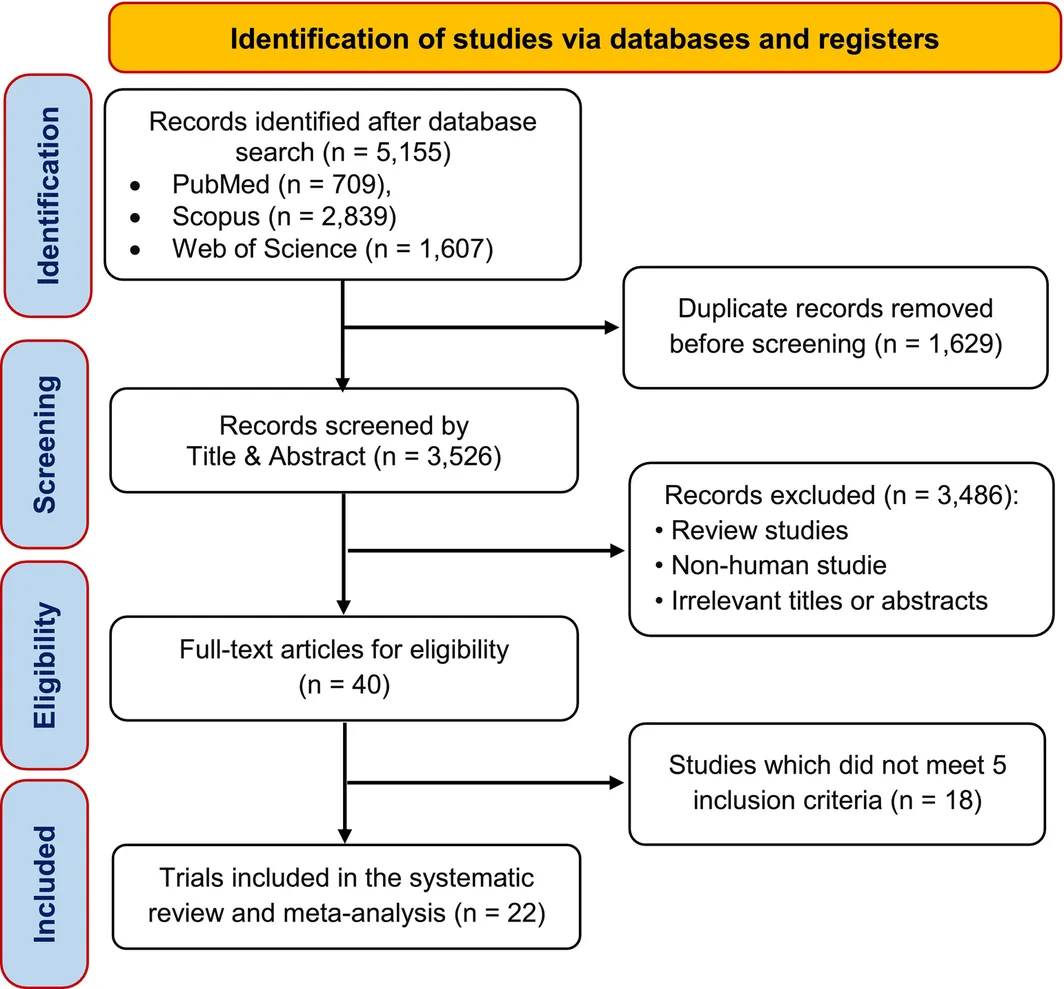
PRISMA flowchart of study selection for inclusion trials in the systematic review.

### 3.2 Study Characteristics

Table 1 shows the baseline characteristics of the included studies. The meta‐analysis included 22 RCTs (comprising 33 intervention arms) (Leonhardt 1978; Qureshi et al. 1991, 1997, 2002; Wahlqvist et al. 1992; McDowell et al. 1994; Tomeo et al. 1995; Chowienczyk et al. 1998; Mensink et al. 1999; Kugiyama et al. 1999; Heitzer et al. 1999; O'Byrne et al. 2000; Kaikkonen et al. 2000; Borovničar et al. 2000; Mustad et al. 2002; Pereira et al. 2004; Baliarsingh et al. 2005; Sutken et al. 2006; Leonard et al. 2007; Zaiden et al. 2010; Tabaei et al. 2020; Namayandeh et al. 2007), published between 1978 and 2020, involving 1001 participants (532 in the intervention group and 531 in the control group). Participants in crossover studies contributed to both the intervention and control counts; however, they were counted only once in the total population. Geographically, the studies were from diverse regions, including the USA (Qureshi et al. 1997; O'Byrne et al. 2000; Mustad et al. 2002; Leonard et al. 2007), Iran (Tabaei et al. 2020; Namayandeh et al. 2007), Finland (Heitzer et al. 1999; Kaikkonen et al. 2000), India (Baliarsingh et al. 2005), Northern Ireland (McDowell et al. 1994), Sweden (Leonhardt 1978), the UK (Chowienczyk et al. 1998), Slovenia (Borovničar et al. 2000), Japan (Kugiyama et al. 1999), Turkey (Sutken et al. 2006), Malaysia (Tomeo et al. 1995), Singapore (Zaiden et al. 2010), Brazil (Pereira et al. 2004), the Netherlands (Mensink et al. 1999), Pakistan (Qureshi et al. 2002), and Australia (Wahlqvist et al. 1992). Most had a parallel, randomized, placebo‐controlled, double‐blind design, whereas some used crossover designs (Leonhardt 1978; Qureshi et al. 1991; Pereira et al. 2004) or single‐blind methodologies (Leonard et al. 2007). The mean ages ranged from 25 to 78 years, and the baseline BMI ranged from 19 to 39 kg/m^2^. The duration of vitamin E administration ranged from 4 to 77 weeks. The majority of studies enrolled both sexes, except for three studies that included only males (McDowell et al. 1994; Heitzer et al. 1999; Pereira et al. 2004) and two that included only women (Wahlqvist et al. 1992; Tabaei et al. 2020). The intervention group received different doses of vitamin E (55–1043 IU/day), whereas the control group received either a placebo (Leonhardt 1978; Qureshi et al. 1991, 1997, 2002; Wahlqvist et al. 1992; McDowell et al. 1994; Tomeo et al. 1995; Chowienczyk et al. 1998; Mensink et al. 1999; Kugiyama et al. 1999; Heitzer et al. 1999; O'Byrne et al. 2000; Kaikkonen et al. 2000; Borovničar et al. 2000; Mustad et al. 2002; Baliarsingh et al. 2005; Leonard et al. 2007; Zaiden et al. 2010), statins (e.g., atorvastatin, simvastatin, lovastatin) (Pereira et al. 2004; Tabaei et al. 2020; Namayandeh et al. 2007), or gemfibrozil (Sutken et al. 2006). Studies included patients with hypercholesterolemia (Qureshi et al. 1991, 1997, 2002; Wahlqvist et al. 1992; McDowell et al. 1994; Chowienczyk et al. 1998; Heitzer et al. 1999; O'Byrne et al. 2000; Kaikkonen et al. 2000; Borovničar et al. 2000; Mustad et al. 2002; Pereira et al. 2004; Leonard et al. 2007; Zaiden et al. 2010; Namayandeh et al. 2007), hyperlipidemia (Leonhardt 1978; Tomeo et al. 1995; Mensink et al. 1999; Baliarsingh et al. 2005; Sutken et al. 2006; Tabaei et al. 2020), remnant lipoprotein disorders (Kugiyama et al. 1999), coronary artery disease (Chowienczyk et al. 1998), carotid stenosis (Tomeo et al. 1995), and T2DM (Tabaei et al. 2020).

**TABLE 1:** Characteristics of the included trials in the systematic review and meta‐analysis.

Study	Country	Study design	Health status of participant	Sex (M/F)	Sample size (IG/CG)	Trial duration	Age (years)		BMI (kg/m^2^)		Intervention	Control
							IG	CG	IG	CG		
Leonhardt (1978)	Sweden	Crossover, R, PC, DB	Hyperlipidemia	M: 12 F: 5	17 (17/17)	8 weeks	59	59	NR	NR	α‐tocopherol (596 IU/day)	Placebo
Qureshi et al. (1991)	Malaysia	Crossover, R, PC, DB	Hypercholesterolemia	M: 14 F: 11	25 (25/25)	4 weeks	30–60	30–60	NR	NR	Tocovid (298 IU/day)	Placebo
Wahlqvist et al. (1992)	Australia	Parallel, R, PC, DB	Hypercholesterolemia	M: 0 F: 23	23 (11/12)	20 weeks	51.9 ± 6.9	55.5 ± 12.6	29.4 ± 5.7	31.1 ± 5.1	Tocotrienols (α, γ, δ) (89.4 IU/day)	Placebo
McDowell et al. (1994)	Northern Ireland	Parallel, R, PC, DB	Hypercholesterolemia	NR	16 (8/8)	8 weeks	43.8	43.8	NR	NR	α‐tocopherol (400 IU/day)	Placebo
Tomeo et al. (1995)	Malaysia	Parallel, R, PC, DB	Hyperlipidemia + carotid stenosis	M: 23 F: 27	50 (25/25)	77 weeks	66.4	66.7	NR	NR	Tocopherol (α) + tocotrienol (447 IU/day)	Placebo
Qureshi et al. (1997)	USA	Parallel, R, PC, DB	Hypercholesterolemia	M: 19 F: 22	41 (20/21)	12 weeks	41.5 ± 15.5	42.8 ± 14	25.5 ± 6.57	27.3 ± 5	Tocovid (298 IU/day)	Placebo
Chowienczyk et al. (1998)	UK	Parallel, R, PC, DB	Hypercholesterolemia (+CAD)	M: 18 F: 2	20 (10/10)	8 weeks	39–71	39–71	NR	NR	RRR‐α‐tocopheryl acetate (596 IU/day)	Placebo
Mensink et al. (1999)	Netherland	Parallel, R, PC, DB	Hyperlipidemia	M: 40 F: 0	40 (20/20)	6 weeks	47	45	25.1	25.3	Tocotrienol (59.6 IU/day)	Placebo
Kugiyama et al. (1999) (a)	Japan	Parallel, R, PC, DB	High remnant lipoprotein levels	M: 17 F: 23	40 (20/20)	4 weeks	58.3 ± 8.4	59.6 ± 8.4	23.3 ± 2.9	23.4 ± 2.8	α‐tocopherol acetate (300 IU/day)	Placebo
Kugiyama et al. (1999) (b)	Japan	Parallel, R, PC, DB	Low remnant lipoprotein levels	M: 13 F: 17	30 (15/15)	4 weeks	61.7 ± 9.2	60.8 ± 7.6	22.9 ± 3.4	22.8 ± 3.8	α‐tocopherol acetate (300 IU/day)	Placebo
Heitzer et al. (1999) (a)	Finland	Parallel, R, PC, DB	Hypercholesterolemia	M: 13 F: 0	13 (9/4)	16 weeks	56	53	NR	NR	α‐tocopherol acetate (544 IU/day)	Placebo
Heitzer et al. (1999) (b)	Finland	Parallel, R, PC, DB	Hypercholesterolemia + smoking	M: 14 F: 1	15 (10/5)	16 weeks	53	52	NR	NR	α‐tocopherol acetate (555 IU/day)	Placebo
O'Byrne et al. (2000) (a)	USA	Parallel, R, PC, DB	Hypercholesterolemia	M: 11 F: 15	26 (13/13)	8 weeks	40.0 ± 11	38.7 ± 9.7	29.6 ± 6.9	30.0 ± 9.0	Tocotrienyl acetate (550 IU/day)	Placebo
O'Byrne et al. (2000) (b)	USA	Parallel, R, PC, DB	Hypercholesterolemia	M: 12 F: 13	25 (12/13)	8 weeks	43.2 ± 13.2	38.7 ± 9.7	28.8 ± 7.7	30.0 ± 9.0	Tocotrienyl acetate (550 IU/day)	Placebo
O'Byrne et al. (2000) (c)	USA	Parallel, R, PC, DB	Hypercholesterolemia	M: 11 F: 15	26 (13/13)	8 weeks	41.4 ± 9.8	38.7 ± 9.7	28.3 ± 5.8	30.0 ± 9.0	Tocotrienyl acetate (550 IU/day)	Placebo
Kaikkonen et al. (2000) (a)	Finland	Parallel, R, PC, DB	Hypercholesterolemia	M: 8 F: 12	20 (10/10)	12 weeks	60.7 ± 5.7	60.7 ± 5.7	26.9 ± 3.6	26.9 ± 3.6	α‐tocopherol (1043 IU/day)	Placebo
Kaikkonen et al. (2000) (b)	Finland	Parallel, R, PC, DB	Hypercholesterolemia	M: 8 F: 12	20 (10/10)	12 weeks	60.7 ± 5.7	60.7 ± 5.7	26.9 ± 3.6	26.9 ± 3.6	α‐tocopherol (1043 IU/day) + Q10	Q10
Borovničar et al. (2000)	Slovenia	Parallel, R, PC, DB	Hypercholesterolemia	M: 44 F: 0	44 (22/22)	24 weeks	30–60	30–60	NR	NR	α‐tocopherol acetate (596 IU/day)	Placebo
Mustad et al. (2002) (a)	USA	Parallel, R, PC, DB	Hypercholesterolemia	M: 20 F: 14	34 (17/17)	4 weeks	44 ± 8	41 ± 4	26 ± 3.2	28 ± 2.8	Tocotrienols (α‐γ) (298 IU/day)	Placebo
Mustad et al. (2002) (b)	USA	Parallel, R, PC, DB	Hypercholesterolemia	M: 21 F: 13	34 (17/17)	4 weeks	42 ± 8	41 ± 4	26 ± 4	28 ± 2.8	Tocotrienols (α, γ, δ) (298 IU/day)	Placebo
Mustad et al. (2002) (c)	USA	Parallel, R, PC, DB	Hypercholesterolemia	M: 19 F: 14	33 (16/17)	4 weeks	45 ± 8	41 ± 4	27 ± 2.8	28 ± 2.8	Tocotrienol (298 IU/day)	Placebo
Qureshi et al. (2002) (a)	Pakistan	Parallel, R, PC, DB	Hypercholesterolemia	M: 30 F: 6	36 (18/18)	5 weeks	47.3 ± 5.2	48.4 ± 5.8	25.9 ± 4.1	27.5 ± 3.7	Tocotrienol‐rich fraction (55 IU/day)	Control
Qureshi et al. (2002) (b)	Pakistan	Parallel, R, PC, DB	Hypercholesterolemia	M: 30 F: 6	36 (18/18)	5 weeks	49.2 ± 7.8	48.4 ± 5.8	30.3 ± 3.3	27.5 ± 3.7	Tocotrienol‐rich fraction (110 IU/day)	Control
Qureshi et al. (2002) (c)	Pakistan	Parallel, R, PC, DB	Hypercholesterolemia	M: 30 F: 6	36 (18/18)	5 weeks	48.3 ± 6.9	48.4 ± 5.8	25.6 ± 3.6	27.5 ± 3.7	Tocotrienol‐rich fraction (220 IU/day)	Control
Qureshi et al. (2002) (d)	Pakistan	Parallel, R, PC, DB	Hypercholesterolemia	M: 30 F: 6	36 (18/18)	5 weeks	47.9 ± 6.3	48.4 ± 5.8	29.1 ± 3.2	27.5 ± 3.7	Tocotrienol‐rich fraction (440 IU/day)	Control
Pereira et al. (2004)	Brazil	Crossover, R, PC	Hypercholesterolemia	M: 12 F: 13	25 (25/25)	8 weeks	46.2 ± 7.2	46.2 ± 7.2	≥ 30	≥ 30	α‐tocopherol (400 IU/day) + Simvastatin	Simvastatin
Baliarsingh et al. (2005)	India	Parallel, R, PC, DB	Hyperlipidemia	M: 10 F: 9	19 (10/9)	8 weeks	48.5 ± 6.6	52.6 ± 5.9	25.5 ± 2.9	23.2 ± 2.3	Tocotrienols (268.2 IU/day)	Placebo
Sutken et al. (2006) (a)	Turkey	Parallel, R, CO	Hyperlipidemia (Elderly)	M: 24 F: 21	45 (22/23)	4 weeks	72 ± 4.2	71 ± 3	25 ± 3.4	26 ± 6	Vitamin E (894 IU/day) + Gemfibrozil	Gemfibrozil
Sutken et al. (2006) (b)	Turkey	Parallel, R, CO	Hyperlipidemia (Young)	M: 11 F: 11	22 (12/10)	4 weeks	30 ± 4.6	29 ± 5.3	23 ± 3.2	24 ± 2.8	Vitamin E (894 IU/day) + Gemfibrozil	Gemfibrozil
Namayandeh et al. (2007)	Iran	Parallel, R, CO	Hypercholesterolemia	M: 25 F: 35	60 (30/30)	8 weeks	56.9	57	NR	NR	Vitamin E (400 IU/day) + Lovastatin	Lovastatin
Leonard et al. (2007)	USA	Parallel, R, PC, SB	Hypercholesterolemia	M: 23 F: 21	44 (21/23)	12 weeks	66 ± 12	65 ± 11	28 ± 4	28 ± 4	RRR‐ α‐tocopheryl acetate (400 IU/day)	Placebo
Zaiden et al. (2010)	Singapore	Parallel, R, PC, DB	Hypercholesterolemia	M: 14 F: 6	20 (10/10)	8 weeks	43	43	> 25	> 25	Tocotrienol (γ + δ) (178.8 IU/day)	Placebo
Tabaei et al. (2020)	Iran	Parallel, R, PC, DB	Hyperlipidemia (+T2DM)	M: 0 F: 30	30 (15/15)	12 weeks	50.4 ± 1.4	50.6 ± 1.1	26.8 ± 0.5	27.1 ± 0.9	Vitamin E (400 IU/day)	Atorvastatin

*Note:* Different letters (A–D) indicate different intervention arms (effect sizes) reported within the same study.

Abbreviations: CAD, coronary artery disease; Cont., control; DB, double‐blind; F, female; Int., intervention; M, male; NR, not reported; PC, placebo‐controlled; R, randomized clinical trial; SB, single‐blind; T2DM, Type 2 diabetes mellitus; TRF, tocotrienol‐rich fraction.

### 3.3 Quality Assessment

The quality assessment using the Cochrane RoB‐2 tool indicated that most studies had a low risk of bias across the five domains, demonstrating a strong methodological quality. Studies by Leonhardt (1978), Kugiyama et al. (1999), O'Byrne et al. (2000), and Tabaei et al. (2020) were rated as low risk, reflecting robust designs. However, some studies, including Sutken et al. (2006) and Pereira et al. (2004), reported a moderate‐to‐high risk, primarily due to missing data and deviations from intended interventions. Studies like Leonard et al. (2007) and Namayandeh et al. (2007) raised concerns across multiple domains, indicating potential limitations. Despite these issues, most studies exhibited low bias, ensuring the overall reliability of the results in this review (Figure 2).

**FIGURE 2:**
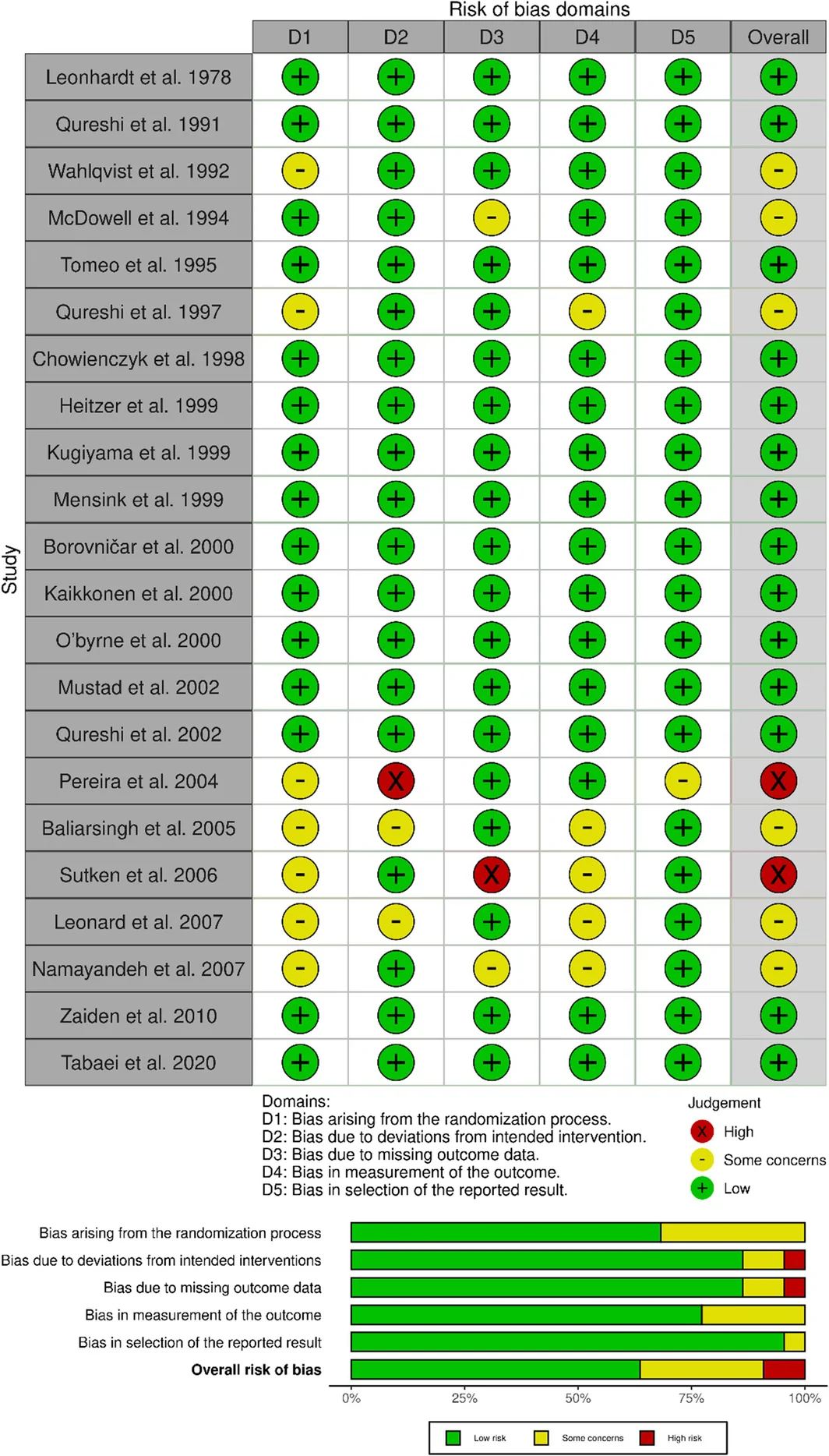
Quality assessment of studies according to Cochrane risk‐of‐bias 2 (RoB‐2).

### 3.4 Meta‐Analysis: Effect of Vitamin E on TG

The pooled analysis of 31 effect sizes from 21 studies (Leonhardt 1978; Qureshi et al. 1991, 1997, 2002; Wahlqvist et al. 1992; McDowell et al. 1994; Tomeo et al. 1995; Chowienczyk et al. 1998; Mensink et al. 1999; Kugiyama et al. 1999; O'Byrne et al. 2000; Kaikkonen et al. 2000; Borovničar et al. 2000; Mustad et al. 2002; Pereira et al. 2004; Baliarsingh et al. 2005; Sutken et al. 2006; Leonard et al. 2007; Zaiden et al. 2010; Tabaei et al. 2020; Namayandeh et al. 2007) demonstrated a clinically and statistically significant decrease in serum TG levels following vitamin E administration in dyslipidemic patients (WMD: −8.14 mg/dL; 95% CI: −15.05 to −1.23; *p* = 0.021), with low heterogeneity among studies (*I* ^2^ = 21.8%, *p* = 0.140) (Figure 3A).

**FIGURE 3:**
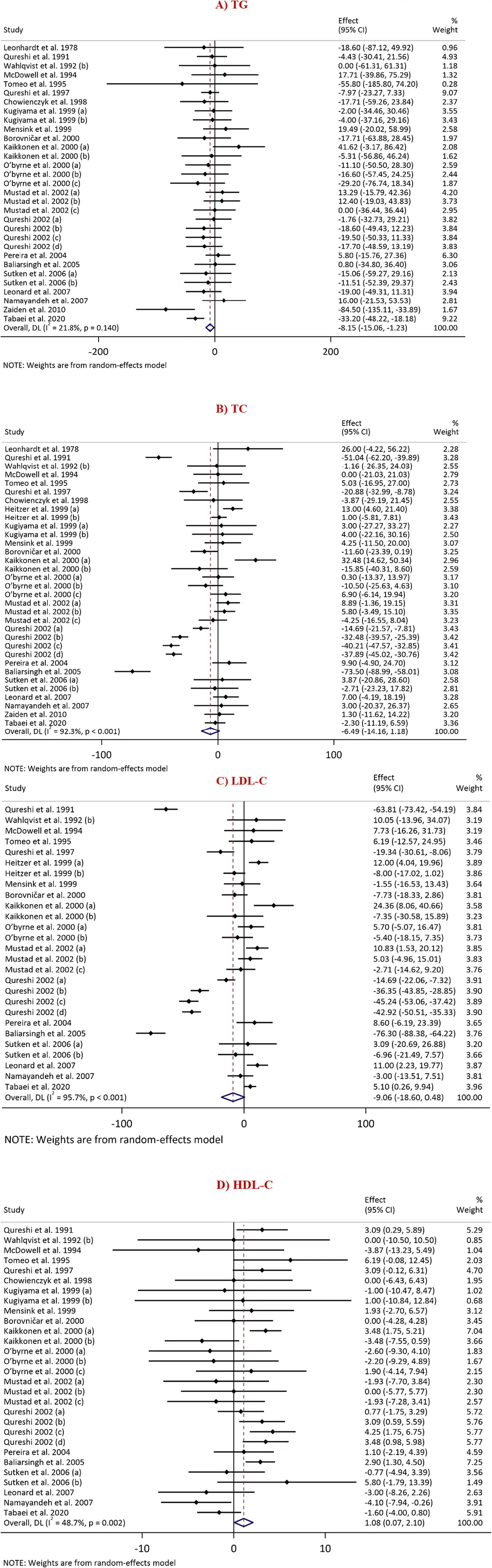
Forest plot demonstrating mean difference (MD) with 95% confidence intervals (CIs) for the effect of the vitamin E administration on (A) triglycerides (TG), (B) total cholesterol (TC), (C) low‐density lipoprotein cholesterol (HDL‐C), and (D) high‐density lipoprotein cholesterol (LDL‐C) in patients with dyslipidemia.

Subgroup analyses revealed a significant effect in patients with baseline serum TG levels ≥ 150 mg/dL (WMD: −9.46 mg/dL; 95% CI: −16.55 to −2.37; *p* = 0.009). Conversely, no significant increase in TG levels was observed in patients with normal baseline serum TG (< 150 mg/dL). In subgroup analyses, no significant changes were observed regarding trial duration, intervention type (tocopherol vs. tocotrienol), or dosage (≤ 400 IU/day vs. > 400 IU/day). However, overweight individuals (BMI 25–29.9 kg/m^2^) showed a marginally insignificant reduction (WMD: −9.36 mg/dL; *p* = 0.068) (Table 2).

**TABLE 2:** Meta‐analysis findings of vitamin E administration on lipid profile in patients with dyslipidemia.

Meta‐analysis	No. of arms	WMD (95% CI)	*p*	Heterogeneity		
				*p* heterogeneity	*I* ^2^	*p* between subgroups
Effects of vitamin E on triglycerides (TG) (mg/dL)						
Overall effect	31	−8.14 (−15.05, −1.23)	0.021*	0.140	21.8%	
Serum baseline						
< 150 mg/dL	4	4.20 (−21.86, 30.27)	0.752	0.155	42.8%	0.321
≥ 150 mg/dL	27	−9.46 (−16.55, −2.37)	0.009*	0.196	18.6%	
Trial duration						
< 12 weeks	23	−4.92 (−12.16, 2.32)	0.183	0.513	0.0%	0.403
≥ 12 weeks	8	−12.47 (−28.61, 3.66)	0.130	0.066	47.1%	
Intervention type						
Tocopherol	14	−7.24 (−18.97, 4.48)	0.226	0.096	34.9%	0.971
Tocotrienol	16	−7.51 (−16.16, 1.13)	0.089	0.300	13.4%	
Intervention dose (IU/day)						
≤ 400	20	−7.06 (−15.74, 1.62)	0.111	0.034	40.0%	0.697
> 400	11	−10.35 (−24.51, 3.79)	0.151	0.754	0.0%	
Baseline BMI (kg/m^2^)						
Normal (18.5–24.9)	3	−5.05 (−25.23, 15.11)	0.623	0.935	0.0%	0.871
Overweight (25–29.9)	19	−9.36 (−19.43, 0.69)	0.068	0.024	43.1%	
Obese (> 30)	2	−3.81 (−27.17, 19.55)	0.749	0.204	38.1%	
Effects of vitamin E on total cholesterol (TC) (mg/dL)						
Overall effect	33	−6.48 (−14.15, 1.17)	0.097	< 0.001	92.3%	
Serum baseline						
≥ 200 mg/dL	31	−7.22 (−15.17, 0.72)	0.075	< 0.001	92.5%	0.038
< 200 mg/dL	2	6.53 (−3.75, 16.82)	0.213	0.836	0.0%	
Trial duration						
< 12 weeks	23	−9.61 (−19.23, 0.01)	0.050	< 0.001	92.6%	0.109
≥ 12 weeks	10	0.76 (−7.51, 9.03)	0.857	< 0.001	77.7%	
Intervention type						
Tocopherol	16	3.90 (−1.61, 9.42)	0.166	0.012	49.8%	0.001
Tocotrienol	16	−16.50 (−27.39, −5.61)	0.003*	< 0.001	94.2%	
Intervention dose (IU/day)						
≤ 400	20	−12.37 (−22.37, −2.38)	0.015*	< 0.001	93.6%	0.013
> 400	13	2.96 (−3.80, 9.72)	0.391	0.002	61.1%	
Baseline BMI (kg/m^2^)						
Normal (18.5–24.9)	3	0.54 (−13.70, 14.79)	0.940	0.910	0.0%	0.589
Overweight (25–29.9)	19	−8.23 (−18.38, 1.90)	0.112	< 0.001	93.1%	
Obese (> 30)	2	−11.80 (−53.32, 29.71)	0.577	< 0.001	96.1%	
Effects of vitamin E on low‐density lipoprotein cholesterol (LDL‐C) (mg/dL)						
Overall effect	27	−9.06 (−18.60, 0.47)	0.063	< 0.001	95.7%	
Serum baseline						
≥ 130 mg/dL	25	−10.48 (−20.69, −0.27)	0.044*	< 0.001	95.3%	0.003
< 130 mg/dL	2	6.87 (1.57, 12.17)	0.011*	0.248	25.0%	
Trial duration						
< 12 weeks	17	−15.6 (−28.56, −2.83)	0.017*	< 0.001	96.0%	0.017
≥ 12 weeks	10	2.27 (−5.07, 9.61)	0.544	< 0.001	78.2%	
Intervention type						
Tocopherol	12	3.21 (−2.18, 8.61)	0.244	0.002	62.5%	0.002
Tocotrienol	14	−20.19 (−34.23, −6.15)	0.005*	< 0.001	96.4%	
Intervention dose (IU/day)						
≤ 400	17	−15.03 (−28.05, −2.01)	0.024*	< 0.001	96.9%	0.029
> 400	10	1.45 (−5.50, 8.40)	0.683	0.003	64.4%	
Baseline BMI (kg/m^2^)						
Normal (18.5–24.9)	1	−6.96 (−21.49, 7.56)	0.348	—	—	0.947
Overweight (25–29.9)	17	−8.67 (−20.83, 3.49)	0.162	< 0.001	95.9%	
Obese (> 30)	2	−14.34 (−58.38, 29.69)	0.523	< 0.001	96.5%	
Effects of vitamin E on high‐density lipoprotein cholesterol (HDL‐C) (mg/dL)						
Overall effect	29	1.08 (0.06, 2.10)	0.037*	0.002	48.7%	
Serum baseline HDL‐C						
≥ 40 mg/dL	21	0.28 (−1.01, 1.57)	0.669	0.004	50.9%	0.014
< 40 mg/dL	8	2.67 (1.27, 4.08)	< 0.001*	0.227	25.3%	
Trial duration						
< 12 weeks	21	1.40 (0.32, 2.48)	0.011*	0.065	34.0%	0.559
≥ 12 weeks	8	0.61 (−1.80, 3.02)	0.618	0.001	70.2%	
Intervention type						
Tocopherol	13	−0.46 (−2.33, 1.40)	0.626	0.005	58.1%	0.007
Tocotrienol	15	2.39 (1.51, 3.26)	< 0.001*	0.386	5.9%	
Intervention dose (IU/day)						
≤ 400	19	1.17 (−0.01, 2.36)	**0.054**	0.007	50.2%	0.774
> 400	10	0.81 (−1.35, 2.97)	0.464	0.031	51.0%	
Baseline BMI (kg/m^2^)						
Normal (18.5–24.9)	3	2.71 (−2.58, 8.01)	0.316	0.520	0.0%	0.453
Overweight (25–29.9)	18	0.96 (−0.28, 2.22)	0.131	0.003	55.0%	
Obese (> 30)	2	2.36 (0.37, 4.35)	0.020*	0.345	0.0%	

*Note:* Bold values with asterisk (*) indicate statistical significance (*p* value < 0.05).

Abbreviations: BMI, body mass index; CI, confidence interval; HDL‐C, high‐density lipoprotein cholesterol; LDL‐C, low‐density lipoprotein cholesterol; TC, total cholesterol; TG, triglycerides; WMD, weighted mean difference.

### 3.5 Meta‐Analysis: Effect of Vitamin E on TC

The overall random‐effect pooled analysis of 33 effect sizes from included studies revealed that vitamin E administration was associated with a nonsignificant decrease in TC levels (WMD: −6.48 mg/dL; 95% CI: −14.15 to 1.17; *p* = 0.097), with high heterogeneity (*I* ^2^ = 92.3%, *p* < 0.001) among studies (Leonhardt 1978; Qureshi et al. 1991, 1997, 2002; Wahlqvist et al. 1992; McDowell et al. 1994; Tomeo et al. 1995; Chowienczyk et al. 1998; Mensink et al. 1999; Kugiyama et al. 1999; Heitzer et al. 1999; O'Byrne et al. 2000; Kaikkonen et al. 2000; Borovničar et al. 2000; Mustad et al. 2002; Pereira et al. 2004; Baliarsingh et al. 2005; Sutken et al. 2006; Leonard et al. 2007; Zaiden et al. 2010; Tabaei et al. 2020; Namayandeh et al. 2007) (Figure 3B).

Subgroup analysis indicated a marginally significant decrease in patients with baseline serum TC ≥ 200 mg/dL (WMD: −7.22 mg/dL; *p* = 0.075), whereas those with normal TC (< 200 mg/dL) showed no treatment effect. Among vitamin E isoforms, tocotrienol formulations produced significant reductions in TC (WMD: −16.50 mg/dL; 95% CI: −27.39 to −5.61; *p* = 0.003), whereas tocopherol showed no significant benefit. Dosage analysis revealed that lower doses (≤ 400 IU/day) were associated with a significant decrease (WMD: −12.37 mg/dL; 95% CI: −22.37 to −2.38; *p* = 0.015), whereas higher doses were ineffective (Table 2).

### 3.6 Meta‐Analysis: Effect of Vitamin E on LDL‐C

The random‐effects pooled analysis of 27 effect sizes from 18 studies showed that vitamin E administration was associated with a statistically nonsignificant decrease in LDL‐C levels of uncertain clinical relevance (WMD: −9.06 mg/dL; 95% CI: −18.60 to 0.47; *p* = 0.063), with a highly significant heterogeneity among studies (*I* ^2^ = 95.7%, *p* < 0.001) (Qureshi et al. 1991, 1997, 2002; Wahlqvist et al. 1992; McDowell et al. 1994; Tomeo et al. 1995; Mensink et al. 1999; Heitzer et al. 1999; O'Byrne et al. 2000; Kaikkonen et al. 2000; Borovničar et al. 2000; Mustad et al. 2002; Pereira et al. 2004; Baliarsingh et al. 2005; Sutken et al. 2006; Leonard et al. 2007; Tabaei et al. 2020; Namayandeh et al. 2007) (Figure 3C).

Subgroup analyses indicated that patients with elevated baseline serum LDL‐C (≥ 130 mg/dL) experienced a significant reduction (WMD: −10.48 mg/dL; 95% CI: −20.69 to −0.27; *p* = 0.044), whereas individuals with normal LDL‐C (< 130 mg/dL) unexpectedly exhibited an increase (WMD: 6.87 mg/dL; *p* = 0.011). Notable reductions in LDL‐C were also observed in short‐term interventions (< 12 weeks) (WMD: −15.63 mg/dL; 95% CI: −28.56 to −2.83; *p* = 0.017) and with tocotrienol supplementation (WMD: −20.19 mg/dL; *p* = 0.005). Additionally, lower doses (≤ 400 IU/day) were associated with a significant decrease in LDL‐C (WMD: −15.03 mg/dL; 95% CI: −28.05 to −2.01; *p* = 0.024), whereas higher doses did not show a measurable benefit (Table 2).

### 3.7 Meta‐Analysis: Effect of Vitamin E on HDL‐C

The overall pooled analysis of 29 effect sizes from 19 included studies revealed a significant and beneficial increase in HDL‐C levels (WMD: 1.08 mg/dL; 95% CI: 0.06–2.10; *p* = 0.037) following vitamin E administration in patients with dyslipidemia. In addition, moderate heterogeneity (*I* ^2^ = 48.7%, *p* = 0.002) was observed among studies (Qureshi et al. 1991, 1997, 2002; Wahlqvist et al. 1992; McDowell et al. 1994; Tomeo et al. 1995; Chowienczyk et al. 1998; Mensink et al. 1999; Kugiyama et al. 1999; O'Byrne et al. 2000; Kaikkonen et al. 2000; Borovničar et al. 2000; Mustad et al. 2002; Pereira et al. 2004; Baliarsingh et al. 2005; Sutken et al. 2006; Leonard et al. 2007; Tabaei et al. 2020; Namayandeh et al. 2007) (Figure 3D).

In the subgroup analysis, patients with low baseline HDL‐C (< 40 mg/dL) experienced a numerically greater increase (WMD: 2.67 mg/dL; 95% CI: 1.27–4.08; *p* < 0.001), whereas those with normal HDL‐C (≥ 40 mg/dL) showed no significant change. Among the vitamin E isoforms, tocotrienols were associated with a substantial increase in HDL‐C (WMD: 2.39 mg/dL; 95% CI: 1.51–3.26; *p* < 0.001), whereas tocopherols had no significant effect. Notably, obese patients with dyslipidemia (BMI ≥ 30 kg/m^2^) also demonstrated a significant increase (WMD: 2.36 mg/dL; 95% CI: 0.37–4.35; *p* = 0.020). Furthermore, the improvement in HDL‐C was more pronounced in short‐term interventions (WMD: 1.40 mg/dL; *p* = 0.011) (Table 2).

### 3.8 Linear Meta‐Regression Analysis

Linear meta‐regression showed no significant associations between vitamin E dose or intervention duration and changes in TG, TC, or HDL‐C (all *p* > 0.05), although borderline associations were observed for TC with both dose (*p* = 0.069) and duration (*p* = 0.067). In contrast, intervention duration was significantly associated with changes in LDL‐C (coefficient = 0.08, *p* = 0.043), whereas dose was not (*p* = 0.110) (Table 3, Figure 4).

**TABLE 3:** Linear meta‐regression and nonlinear dose‐ and duration‐response analysis.

Outcomes	Linear meta‐regression analysis				Nonlinear analysis			
	Dose (mg/day)		Duration (week)		Dose (mg/day)		Duration (week)	
	Coefficient	*p*	Coefficient	*p*	Coefficient	*p*	Coefficient	*p*
TG	1.04	0.626	−0.06	0.700	−0.348	0.140	−7.00	0.626
TC	3.83	0.069	0.09	0.067	−163.14	**0.043**	−15.04	0.221
LDL‐C	3.52	0.110	0.08	**0.043**	−197.55	0.050	−26.16	0.087
HDL‐C	−5.26	0.770	1.39	0.121	−0.74	0.324	4.85	**0.033**

*Note:* Bold values indicate statistical significance (*p* value < 0.05).

Abbreviations: HDL‐C, high‐density lipoprotein cholesterol; LDL‐C, low‐density lipoprotein cholesterol; TC, total cholesterol; TG, triglycerides.

**FIGURE 4:**
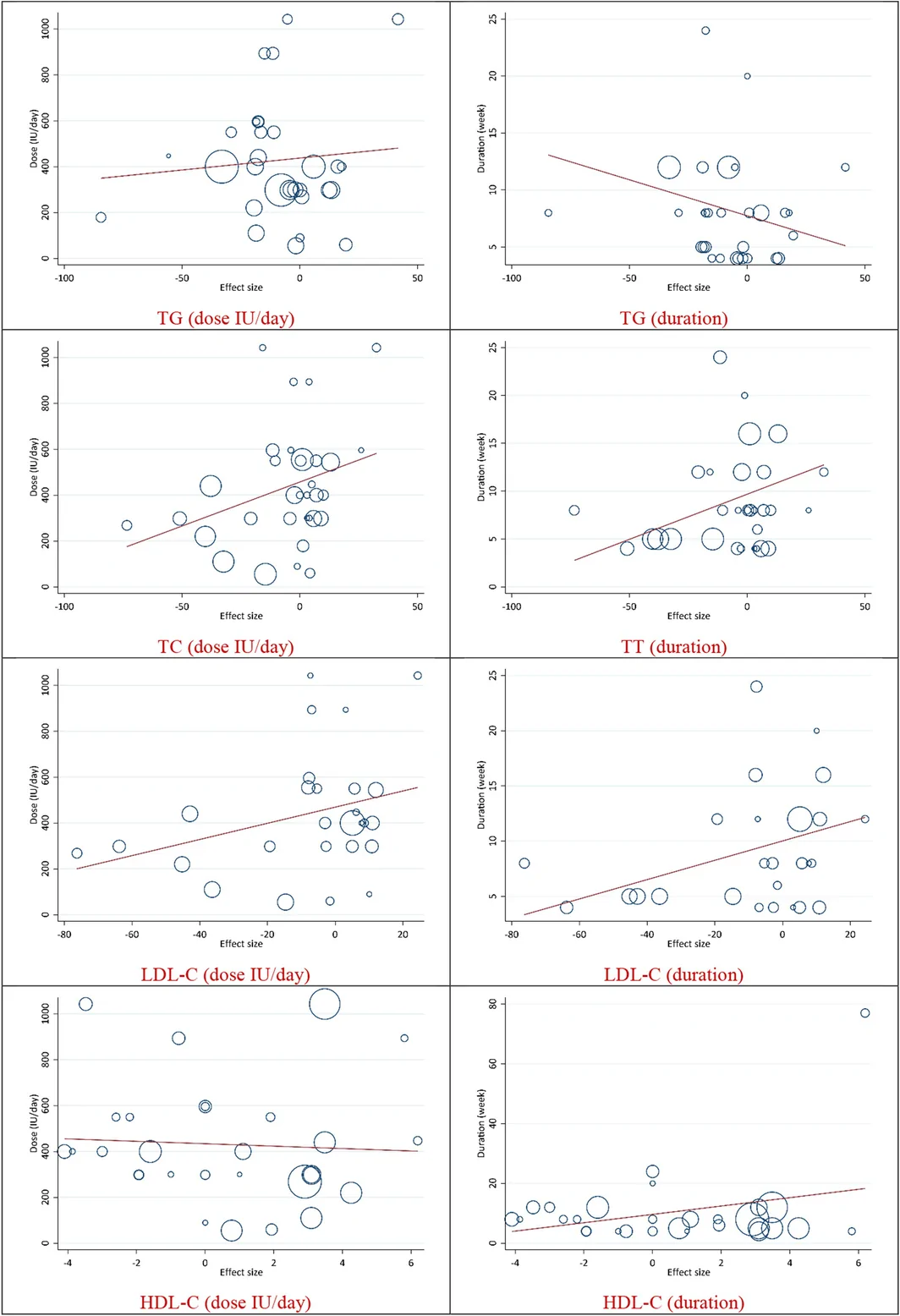
Linear dose–response relations between vitamin E administration and absolute mean differences (MD). Dose–response relations between dose (IU/day) and duration (weeks) of intervention and absolute mean differences in triglycerides (TG), total cholesterol (TC), low‐density lipoprotein cholesterol (LDL‐C), and high‐density lipoprotein cholesterol (HDL‐C) in patients with dyslipidemia.

### 3.9 Nonlinear Analysis

Nonlinear analyses demonstrated a significant inverse dose–response relationship for TC (coefficient = −163.14, *p* = 0.043) and a borderline significant association for LDL‐C (coefficient = −197.55, *p* = 0.050), suggesting greater lipid‐lowering effects at higher vitamin E doses. A significant positive nonlinear association was observed between intervention duration and HDL‐C (coefficient = 4.85, *p* = 0.033), while no significant nonlinear relationships were detected for the remaining outcomes (Table 3, Figure 5).

**FIGURE 5:**
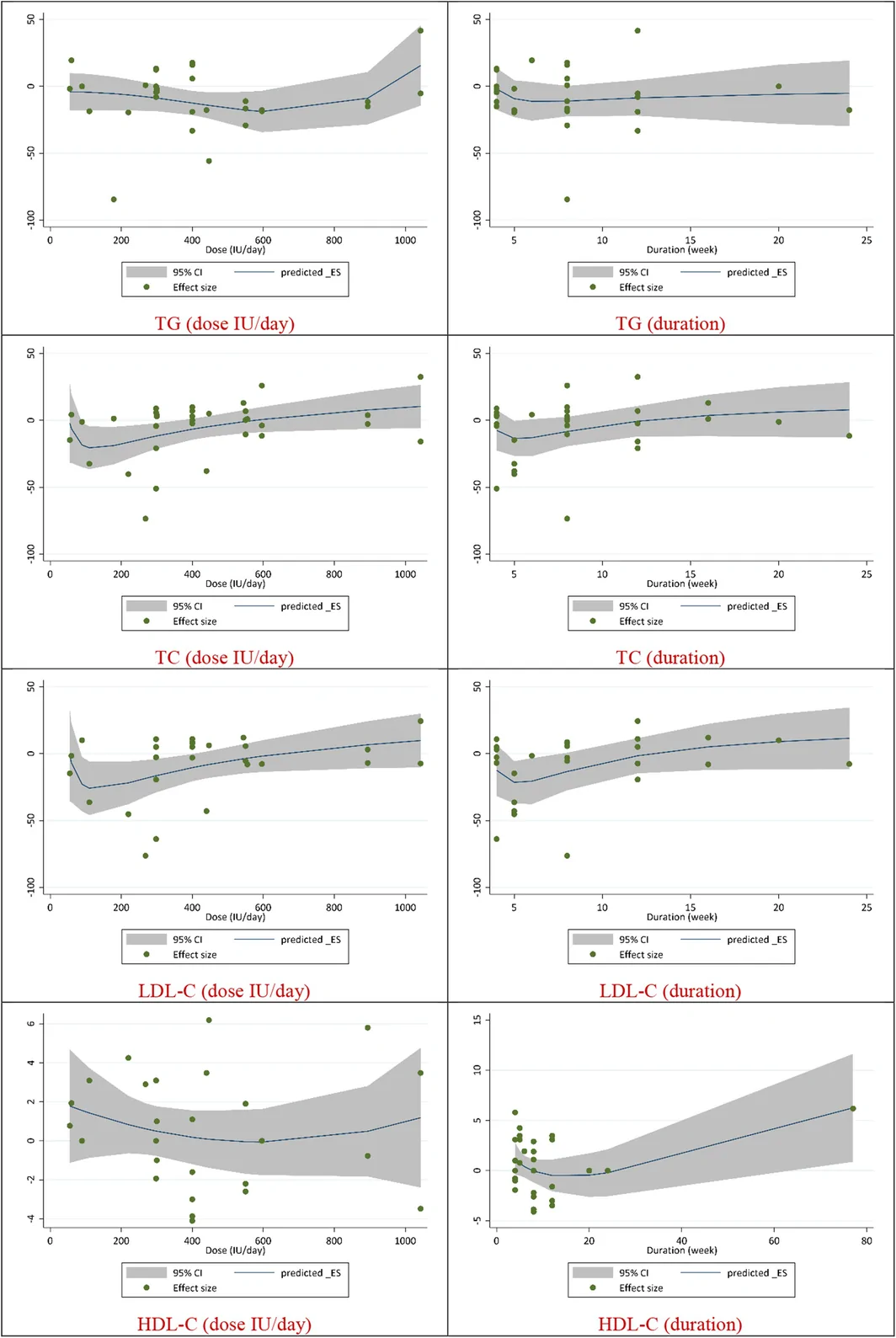
Nonlinear dose–response relations between vitamin E administration and absolute mean differences (MD). Dose–response relations between dose (IU/day) and duration (weeks) of intervention and absolute mean differences in triglycerides (TG), total cholesterol (TC), low‐density lipoprotein cholesterol (LDL‐C), and high‐density lipoprotein cholesterol (HDL‐C) in patients with dyslipidemia.

### 3.10 Publication Bias

Publication bias was evaluated using Begg's and Egger's tests. No significant publication bias was observed for TG, TC, or LDL‐C. In contrast, Egger's test indicated potential publication bias for HDL‐C (*p* = 0.013), although Begg's test did not confirm this finding (*p* = 0.237). Furthermore, the funnel plot exhibited asymmetry, supporting the inference of publication bias, particularly for HDL‐C (Figure 6, Table 4). Because Egger's test indicated potential publication bias for HDL‐C, the trim‐and‐fill method was subsequently performed. The adjusted pooled estimate remained comparable to the primary analysis and did not materially alter the statistical significance or overall interpretation of the findings.

**FIGURE 6:**
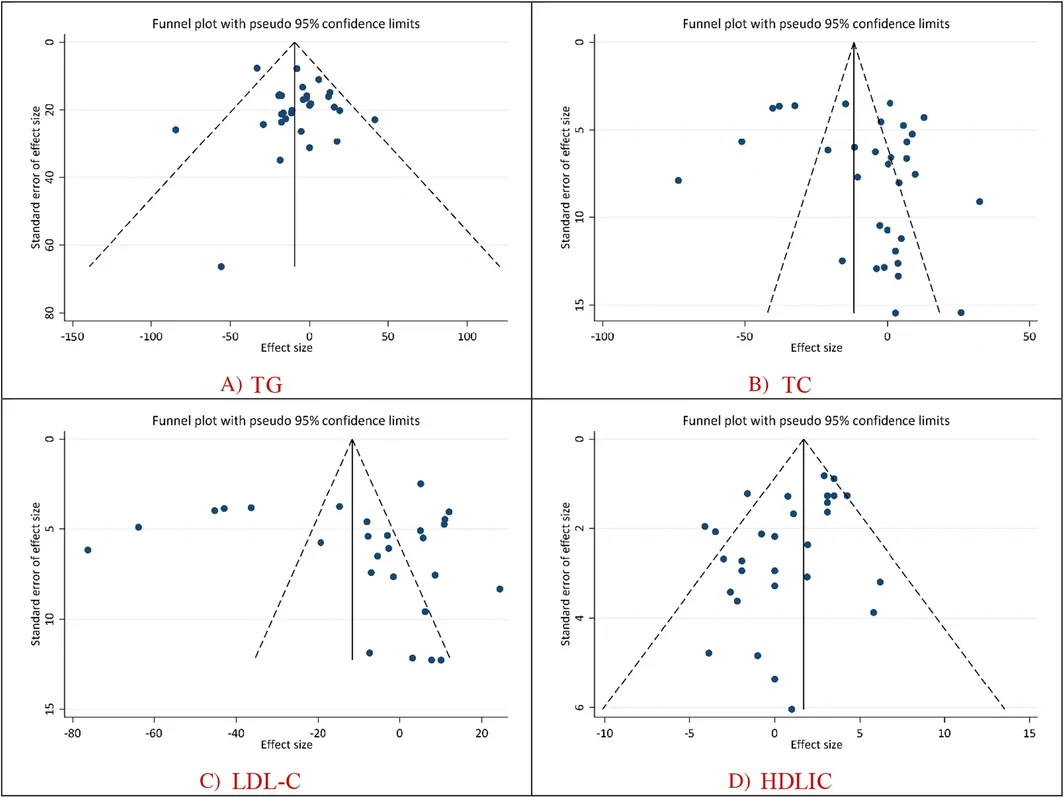
Funnel plots with 95% confidence intervals (CIs) demonstrating publication bias for the effect of vitamin E administration on (A) triglycerides (TG), (B) total cholesterol (TC), (C) low‐density lipoprotein cholesterol (LDL‐C), and (D) high‐density lipoprotein cholesterol (HDL‐C) in patients with dyslipidemia.

**TABLE 4:** Publication bias (*p* value by Begg's and Egger's test) and sensitivity analysis.

Outcomes	Publication bias		Sensitivity analysis
	Begg's test	Egger's test	
TG	0.529	0.444	Tabaei et al. (2020): (WMD: −5.42 mg/dL, 95% CI: −11.61 to 0.76)
TC	0.816	0.096	Kaikkonen et al. (2000) (a): (WMD: −7.70 mg/dL, 95% CI: −15.33 to −0.08)
LDL‐C	0.967	0.700	Heitzer et al. (1999) (a): (WMD: −9.91 mg/dL, 95% CI: −19.65 to −0.17) Kaikkonen et al. (2000) (a): (WMD: −10.31 mg/dL, 95% CI: −19.95 to −0.66) Mustad et al. (2002) (a): (WMD: −9.85 mg/dL, 95% CI: −19.62 to −0.09) Leonard et al. (2007): (WMD: −9.86 mg/dL, 95% CI: −19.63 to −0.10)
HDL‐C	0.237	**0.013**	Qureshi et al. (1991): (WMD: 0.95 mg/dL, 95% CI: −0.10 to 2.01) Tomeo et al. (1995): (WMD: 0.98 mg/dL, 95% CI: −0.03 to 2.00) Qureshi et al. (1997): (WMD: 0.97 mg/dL, 95% CI: −0.08 to 2.02) Mensink et al. (1999): (WMD: 1.04 mg/dL, 95% CI: −0.00 to 2.09) Kaikkonen et al. (2000) (a): (WMD: 0.90 mg/dL, 95% CI: −0.15 to 1.95) Qureshi et al. (2002) (b): (WMD: 0.94 mg/dL, 95% CI: −0.11 to 2.01) Qureshi et al. (2002) (c): (WMD: 0.90 mg/dL, 95% CI: −0.12 to 1.93) Qureshi et al. (2002) (d): (WMD: 0.93 mg/dL, 95% CI: −0.12 to 1.98) Baliarsingh et al. (2005): (WMD: 0.91 mg/dL, 95% CI: −0.16 to 2.00) Sutken et al. (2006) (b): (WMD: 1.01 mg/dL, 95% CI: −0.01 to 2.03)

*Note:* Bold value indicates statistical significance (*p* value < 0.05). The (a), (b), (c), indicates the various intervention arms (effect size) of the study.

Abbreviations: HDL‐C, high‐density lipoprotein cholesterol; LDL‐C, low‐density lipoprotein cholesterol; TC, total cholesterol; TG, triglycerides.

### 3.11 Sensitivity Analysis

Leave‐one‐out sensitivity analyses showed that the overall findings were generally robust, as sequential exclusion of individual studies did not substantially alter the magnitude or direction of the pooled effect estimates (Table 4). For TG, exclusion of Tabaei et al. (2020) attenuated the pooled effect from −8.14 to −5.42 mg/dL (95% CI: −11.61 to 0.76), resulting in loss of statistical significance. Likewise, for TC, omission of Kaikkonen et al. (2000) reduced the pooled estimate to −7.70 mg/dL (95% CI: −15.33 to −0.08), although the effect remained statistically significant. For LDL‐C, exclusion of Heitzer et al. (1999), Kaikkonen et al. (2000), Mustad et al. (2002), or Leonard et al. (2007) yielded pooled WMDs ranging from −9.85 to −10.31 mg/dL, with all estimates remaining statistically significant, indicating that the LDL‐C‐lowering effect was not driven by any single study. In contrast, for HDL‐C, omitting several individual studies yielded pooled WMDs ranging from 0.90 to 1.04 mg/dL, with all corresponding 95% confidence intervals including the null value, consistent with the overall nonsignificant effect. Overall, these findings indicate that the pooled estimates for TC and LDL‐C were robust, whereas the statistical significance of the TG outcome was influenced by the exclusion of Tabaei et al. (2020). No evidence suggested that any single study substantially changed the direction of the pooled effects.

### 3.12 GRADE Assessment

The certainty of the evidence, assessed using the GRADE approach, varied across the lipid outcomes evaluated (Table 5). The evidence supporting the effect of vitamin E on TG was rated as high certainty, with no serious concerns regarding risk of bias, inconsistency, indirectness, imprecision, or publication bias. In contrast, the certainty of evidence for TC and LDL‐C was rated as very low due to very serious inconsistency (high heterogeneity) and serious imprecision, given the absence of statistically significant effects. The evidence for HDL‐C was judged to be of moderate certainty, downgraded by one level due to moderate heterogeneity. Overall, the GRADE assessment indicates high confidence in the TG findings, moderate confidence for HDL‐C, and limited certainty for the effects of vitamin E on TC and LDL‐C.

**TABLE 5:** GRADE assessment of vitamin E administration on serum lipid profile in patients with dyslipidemia.

Outcomes	Risk of bias	Inconsistency	Indirectness	Imprecision	Publication bias	Pooled ES (95% CI)	Quality of evidence
TG	No serious limitation	No serious limitation	No serious limitation	No serious limitation	No serious limitation	−8.14 (−15.05, −1.23)	⊕⊕⊕⊕ High
TC	No serious limitation	Very serious limitation^a^	No serious limitation	Serious limitation^c^	No serious limitation	−6.48 (−14.15, 1.17)	⊕◯◯◯ Very low
LDL‐C	No serious limitation	Very serious limitation^a^	No serious limitation	Serious limitation^c^	No serious limitation	−9.06 (−18.60, 0.47)	⊕◯◯◯ Very low
HDL‐C	No serious limitation	Serious limitation^b^	No serious limitation	No serious limitation	No serious limitation	1.08 (0.06, 2.10)	⊕ ⊕ ⊕◯ Moderate

Abbreviations: HDL‐C, high‐density lipoprotein cholesterol; LDL‐C, low‐density lipoprotein cholesterol; TC, total cholesterol; TG, triglycerides.

^a^ There is a high level of heterogeneity (*I* ^2^ > 75%).

^b^ There is a moderate level of heterogeneity (*I* ^2^ > 40%).

^c^ There is no evidence of significant effects of vitamin E.

## 4 Discussion

### 4.1 Aim and Findings

The present meta‐analysis demonstrated statistically significant improvements in TG and HDL‐C following vitamin E supplementation; however, the magnitude of these effects was relatively modest (approximately 8 mg/dL reduction in TG and 1 mg/dL increase in HDL‐C). Although statistically significant improvements were observed for TG and HDL‐C, the absolute effect sizes were modest and may not translate into clinically meaningful reductions in cardiovascular risk when used as monotherapy. Therefore, statistical significance should not be interpreted as evidence of substantial clinical benefit. Rather, vitamin E supplementation may serve as an adjunctive nutritional intervention for carefully selected patients, whereas established lipid‐lowering therapies and lifestyle modifications remain the cornerstones of dyslipidemia management.

Subgroup analyses indicated that the lipid‐modifying effects of vitamin E varied according to baseline lipid status, intervention duration, vitamin E isoform, dose, and BMI. TG reduction was significant among individuals with baseline TG ≥ 150 mg/dL, whereas TC reduction was observed only with tocotrienol supplementation at doses ≤ 400 IU/day. LDL‐C lowering was significant in participants with baseline LDL‐C ≥ 130 mg/dL, shorter intervention durations, tocotrienol administration, and doses ≤ 400 IU/day. Likewise, HDL‐C improvement was more evident among individuals with baseline HDL‐C < 40 mg/dL and those with obesity. These findings suggest that specific subgroups may derive greater benefit from vitamin E supplementation, particularly tocotrienol formulations; however, given the modest overall effect sizes, vitamin E should be viewed as a complementary rather than primary lipid‐lowering strategy.

Considerable heterogeneity was observed for TC and LDL‐C outcomes, indicating substantial between‐study variability. Although subgroup and influence analyses showed generally consistent findings, residual heterogeneity persisted. This variability likely reflects differences in participant characteristics, baseline lipid concentrations, vitamin E isoforms, intervention dose and duration, concomitant lipid‐lowering therapies, and study methodology. Because the number of available studies was insufficient to support a reliable multivariable meta‐regression, residual heterogeneity could not be fully explained and should be considered when interpreting the pooled estimates.

The apparent discrepancy between subgroup and dose–response analyses likely reflects the different statistical assumptions underlying these methods rather than a true biological contradiction. Subgroup analyses compare predefined categorical dose ranges and may be disproportionately influenced by the characteristics of studies within each category, whereas dose–response models evaluate dose continuously across the entire exposure spectrum. Consequently, the observed inverse nonlinear association suggests that higher doses may provide additional benefit under certain conditions, whereas the subgroup findings indicate that the most consistent evidence currently derives from studies using doses of ≤ 400 IU/day. Differences in vitamin E isoforms, sample‐size distributions, baseline lipid concentrations, and the limited number of trials at higher doses probably contributed to these findings. Therefore, both analyses should be considered complementary rather than conflicting.

Considerable statistical heterogeneity was observed for TC and LDL‐C (*I* ^2^ > 90%), indicating substantial between‐study variability. Nevertheless, pooling was considered appropriate because all studies evaluated oral vitamin E supplementation in adults with dyslipidemia using comparable lipid outcomes, and a random‐effects model was prespecified to account for between‐study variability. Additional exploratory analyses, including subgroup analyses, meta‐regression, and leave‐one‐out sensitivity analyses, identified several potential contributors, including baseline lipid status, vitamin E isoform, intervention duration, dose, BMI, and concomitant therapies. However, these factors explained only part of the observed heterogeneity, suggesting that unmeasured clinical and methodological differences also contributed. Therefore, the pooled estimates should be interpreted cautiously, with greater emphasis placed on the direction and consistency of effects rather than their precise magnitude.

Although all included participants presented with dyslipidemia or lipid abnormalities, the underlying clinical conditions varied considerably and included isolated hypercholesterolemia, hyperlipidemia, Type 2 diabetes mellitus, carotid stenosis, remnant lipoprotein disorders, and coronary artery disease. These conditions differ in their underlying metabolic disturbances, inflammatory burden, oxidative stress, endothelial dysfunction, and concomitant pharmacological management, all of which may influence responsiveness to vitamin E supplementation. Consequently, the pooled estimates should be interpreted as average effects across clinically heterogeneous populations rather than disease‐specific treatment effects. Although subgroup analyses based on lipid concentrations partially addressed this variability, residual clinical heterogeneity likely contributed to the observed between‐study differences, particularly for TC and LDL‐C.

### 4.2 Underlying Mechanism

The modest improvements in lipid parameters observed in the present meta‐analysis may be biologically plausible given the established antioxidant properties of vitamin E. Vitamin E acts primarily by scavenging lipid peroxyl radicals, thereby interrupting the propagation phase of lipid peroxidation and protecting circulating lipoproteins, particularly LDL particles, from oxidative modification (Niki 2021; Porter 1986). Reduced oxidative stress may contribute to improved lipid homeostasis and lower atherosclerotic risk, as supported by evidence demonstrating reductions in oxidative stress biomarkers following vitamin E supplementation (Bergin et al. 2021; Cervantes Gracia et al. 2017). Experimental studies have also suggested that certain vitamin E isoforms, particularly tocotrienols, may inhibit HMG‐CoA reductase activity, providing a potential explanation for the greater lipid‐lowering effects observed with tocotrienol supplementation in our subgroup analyses (Napoli et al. 1998). Nevertheless, these mechanisms are derived largely from experimental and mechanistic studies rather than the present meta‐analysis and should therefore be interpreted as plausible biological explanations rather than direct evidence of causality.

### 4.3 Comparison With Literature

This vitamin exists in two forms, tocopherol and tocotrienol, each with four subforms (α, β, γ, and δ). Tocotrienol isomers have greater antioxidant capacity than tocopherols, and given the growing body of knowledge about their properties, their supplementation has been recognized as a means of achieving therapeutic doses (Ahsan et al. 2014). This may be due to greater distribution within cell membranes and more effective interactions with lipid peroxyl radicals (Wong and Radhakrishnan 2012). However, they are less orally bioavailable compared to tocopherols (Packer et al. 2001). Our subgroup findings suggest a potential trend toward greater lipid‐modifying effects with tocotrienols relative to tocopherols; however, because these comparisons were based on indirect, between‐study subgroup analyses rather than direct, head‐to‐head randomized trials comparing the two isoforms, they should be interpreted as exploratory and hypothesis‐generating rather than conclusive evidence of isoform superiority. Notably, another study published in 2020 and titled “Effects of Tocotrienol Supplementation on Lipid Profile,” found that tocotrienol supplementation did not reduce LDL‐C, TC, or TG concentrations but was considered a candidate for increasing HDL‐C levels (Zuo et al. 2020), illustrating the inconsistency of isoform‐specific findings across the literature. Taken together, although these associations exhibit certain tendencies, their clinical and comparative significance remains to be confirmed in adequately powered, direct comparative trials.

Our study demonstrated a significant decrease in TC and LDL‐C, accompanied by an increase in HDL‐C, within a shorter experimental period (< 12 weeks). A study examining this finding in the DM population found that vitamin E did not affect blood lipid parameters. However, they demonstrated that this vitamin might have beneficial effects on TC and HDL‐C levels when supplemented for ≥ 12 weeks (Mohammad et al. 2021). This discrepancy in results may be due to differences in the populations studied.

A meta‐analysis evaluating vitamin E alone and in combination with other supplements across diverse populations reported inconsistent effects with vitamin E monotherapy; however, when combined with omega‐3 fatty acids, significant reductions in LDL‐C and TG were observed in overweight individuals, with additional TG‐lowering effects noted in patients with diabetes (Mohseni et al. 2021). In contrast, another meta‐analysis found that vitamin E combined with omega‐3 fatty acids had no significant effect on TG, TC, HDL‐C, or LDL‐C, although it was associated with a significant reduction in VLDL levels, suggesting potential but variable benefits of combination therapy (Sepidarkish et al. 2019). Furthermore, evidence from related metabolic conditions supports broader metabolic effects of vitamin E, as an umbrella review of RCTs in NAFLD demonstrated improvements in ALT, AST, hepatic steatosis, and fibrosis, with more pronounced antifibrotic effects observed at doses exceeding 600 IU/day or with treatment durations of at least 12 months (Wang et al. 2023). Collectively, these findings suggest that the metabolic impact of vitamin E may be context‐dependent and potentially enhanced in combination therapies or specific clinical settings.

In addition to vitamin E, several studies have examined the effects of other fat‐soluble vitamins, particularly vitamin D, on lipid profiles and cardiometabolic risk markers across diverse populations (Yu et al. 2025; Hu et al. 2025; Zhang et al. 2022). In a recent meta‐analysis, Yu et al. (2025) reported that vitamin D supplementation significantly reduced TG levels in individuals with overweight or obesity, particularly at doses ≥ 50,000 IU/week, whereas no significant effects were observed on LDL‐C, HDL‐C, or TC. Similarly, Hu et al. (2025) found that vitamin D supplementation significantly improved hypertriglyceridemia in overweight or obese individuals but had no significant effect on LDL‐C concentrations. In contrast, Zhang et al. (2022) reported that vitamin D administration significantly reduced TC and HDL‐C levels. Overall, these findings indicate heterogeneous, population‐dependent effects of vitamin D on lipid parameters, with TG appearing to be the most consistently responsive.

### 4.4 Clinical Implications

Vitamin E supplementation may offer modest benefits as an adjunctive nutritional strategy for dyslipidemia, particularly in reducing TG levels, with greater effects observed in specific subgroups and with tocotrienol supplementation. However, the observed improvements in TG and HDL‐C were small and unlikely to translate into clinically meaningful reductions in cardiovascular risk when used as monotherapy. Therefore, vitamin E should be considered only as a complementary intervention alongside lifestyle modification and guideline‐directed lipid‐lowering therapy, rather than a replacement for established treatments.

### 4.5 Strengths and Limitations

This study offers important insights into the effects of Vitamin E on dyslipidemia, underscoring its potential as a therapeutic strategy to improve lipid profiles. A key strength of the research is its robust methodology, careful patient selection, and thorough biochemical evaluations, which enhance the reliability and clinical relevance of the results. Nonetheless, this study has several limitations that warrant consideration. First, substantial clinical and methodological heterogeneity across the included trials included differences in underlying disease populations, baseline lipid abnormalities, intervention duration, and vitamin E dosage. Second, studies evaluated different vitamin E isoforms (tocopherols and tocotrienols), which have distinct pharmacokinetic and biological properties and may differ in lipid‐lowering efficacy. Because isoform comparisons in this meta‐analysis were based on indirect, between‐study subgroup analyses rather than head‐to‐head randomized trials, apparent differences in efficacy between tocotrienols and tocopherols should be interpreted as exploratory and hypothesis‐generating rather than definitive evidence of isoform superiority. Third, several studies included concomitant lipid‐lowering medications such as statins or gemfibrozil, although these therapies were balanced between intervention and control groups, potentially influencing absolute treatment responses. Fourth, the substantial unexplained heterogeneity observed for TC and LDL‐C suggests that additional unmeasured factors may have influenced treatment effects. Consequently, the findings should be interpreted with appropriate caution.

### 4.6 Future Research Directions

Future investigations should aim for larger, multicenter trials with longer follow‐up periods to confirm these outcomes. Additionally, elucidating the molecular mechanisms by which Vitamin E modulates lipid metabolism and examining its synergistic effects with other lipid‐lowering interventions could further clarify its therapeutic potential.

## 5 Conclusion

In conclusion, vitamin E supplementation in adults with dyslipidemia is associated with modest improvements in serum lipid profiles, primarily reductions in TG and increases in HDL‐C. Effects on TC and LDL‐C were more limited and variable, with evidence suggesting greater efficacy of tocotrienol than of tocopherol isoforms. Overall, these findings indicate potential isoform‐specific lipid‐modifying effects; however, the magnitude and consistency of benefits are modest, and further high‐quality trials are required to confirm clinical relevance.

## Author Contributions

**Mehdi Karimi:** conceptualization, investigation, funding acquisition, writing – original draft, writing – review and editing, visualization, validation, software, formal analysis, project administration, resources, supervision, data curation, methodology. **Kimia Kazemi:** writing – original draft, validation, visualization, software, investigation, funding acquisition, methodology, conceptualization. **Fatemeh Ziyafati:** writing – original draft, data curation, software, investigation, funding acquisition, methodology. **Fereshteh Valizadeh:** writing – original draft, software, investigation, funding acquisition, conceptualization. **Niyousha Shirsalimi:** writing – original draft, software, resources. **Sepehr Ramezanipour:** writing – original draft, software, investigation, funding acquisition. **Ali Estifa:** writing – original draft, software, investigation, funding acquisition, conceptualization, methodology, data curation. **Omid Asbaghi:** formal analysis, software, data curation, supervision, resources, project administration, validation, methodology, visualization, investigation, funding acquisition, conceptualization.

## Funding

The authors have nothing to report.

## Ethics Statement

The authors have nothing to report.

## Conflicts of Interest

The authors declare no conflicts of interest.

## Data Availability

All data used in this meta‐analysis were extracted from published studies. The datasets supporting the findings of this study are available from the original sources cited in the manuscript. Additional information can be provided by the corresponding author upon reasonable request.
